# Dietary Intake Estimates and Urinary Cadmium Levels in Danish Postmenopausal Women

**DOI:** 10.1371/journal.pone.0138784

**Published:** 2015-09-21

**Authors:** Caterina Vacchi-Suzzi, Kirsten T. Eriksen, Keith Levine, Jane McElroy, Anne Tjønneland, Ole Raaschou-Nielsen, James M. Harrington, Jaymie R. Meliker

**Affiliations:** 1 Department of Preventive Medicine and Program in Public Health, Stony Brook University, Stony Brook, New York, United States of America; 2 Danish Cancer Society Research Center, Copenhagen, Denmark; 3 RTI International Trace Inorganics Department, Research Triangle Park, North Carolina, United States of America; 4 Family and Community Medicine, University of Missouri, Columbia, Missouri, United States of America; 5 Department of Environmental Science, Aarhus University, Aarhus, Denmark; Karolinska Institutet, SWEDEN

## Abstract

**Background:**

Cadmium is a known carcinogen that can disrupt endocrine signalling. Cigarette smoking and food are the most common routes of non-occupational exposure to cadmium. Cadmium accumulates in the kidney and can be measured in urine, making urine cadmium (U-Cd) a biomarker of long-term exposure. However dietary-cadmium (D-Cd) intake estimates are often used as surrogate indicator of cadmium exposure in non-smoking subjects. It is therefore important to investigate the concordance between D-Cd estimates obtained with Food Frequency Questionnaires and U-Cd.

**Methods:**

U-Cd levels were compared with estimated dietary-cadmium (D-Cd) intake in 1764 post-menopausal women from the Danish Diet, Cancer and Health cohort. For each participant, a food frequency questionnaire, and measures of cadmium content in standard recipes were used to judge the daily intake of cadmium, normalized by daily caloric intake. Cadmium was measured by ICP-MS in spot urine sampled at baseline and normalized by urinary creatinine. Information on diet, socio-demographics and smoking were self-reported at baseline.

**Results:**

Linear regressions between U-Cd and D-Cd alone revealed minimal but significant positive correlation in never smokers (R^2^ = 0.0076, *β* = 1.5% increase per 1 ng Cd kcal^-1^, p = 0.0085, *n* = 782), and negative correlation in current smokers (R^2^ = 0.0184, *β* = 7.1% decrease per 1 ng Cd kcal^-1^ change, p = 0.0006, *n* = 584). In the full study population, most of the variability in U-Cd was explained by smoking status (R^2^ = 0.2450, *n* = 1764). A forward selection model revealed that the strongest predictors of U-Cd were age in never smokers (Δ R^2^ = 0.04), smoking duration in former smokers (Δ R^2^ = 0.06) and pack-years in current smokers (Δ R^2^ = 0.07). Food items that contributed to U-Cd were leafy vegetables and soy-based products, but explained very little of the variance in U-Cd.

**Conclusions:**

Dietary-Cd intake estimated from food frequency questionnaires correlates only minimally with U-Cd biomarker, and its use as a Cd exposure indicator may be of limited utility in epidemiologic studies.

## Introduction

Exposure to the heavy metal cadmium (Cd) has been linked to increased cancer risk, and is listed as an IARC group 1 carcinogen [1]. A high level of Cd exposure, typical of smelter workers, has been linked to increased risk of multiple diseases [2], and chronic low-level Cd exposure has been implicated in diseases of the kidneys [3] and bone [4, 5]. Cigarette smoking is a primary source of exposure in the non-occupationally exposed population [6]. Non-smokers are exposed to Cd mainly through foodstuff, especially grains and vegetables that acquire the metal from soil, which can be naturally rich in Cd as well as contaminated from anthropomorphic sources such as manure, fertilizers and pesticides [7]. Gender differences in Cd metabolism and toxicity exist, and it is increasingly evident that women tend to absorb more Cd from the intestinal tract [8, 9]. One possible explanation for this phenomenon is that women typically have lower iron and zinc stores than men, which can increase intestinal Cd absorption [10].

Cd has a long half-life (> 10 years) in the human body and tends to accumulate in the liver and kidneys. Therefore, long-term body burden is reflected by urinary Cd levels [11, 12]. Urinary Cd (U-Cd) has been used to assess cancer hazard risk [2]; evidence for increased risk of lung, pancreas, breast and endometrial cancer, as well as for decreased bone density, has been found in relation to elevated U-Cd [13, 14].

Food Frequency Questionnaires (FFQ) are often used in human cohort studies to estimate exposure to nutrients, foodborne xenobiotics, and heavy metals in study populations. Although a handful of studies have been reported that compare U-Cd with dietary Cd (D-Cd) intake estimates from FFQs, employing the average Cd content of a market basket of food items and standard recipes and preparation methods reported for each country [15–18], ours is the largest such study to date. McElroy *et al*. used a limited set of questions about consumption of known Cd-rich food, such as crustaceans and internal organs (liver and kidneys) [19]. Olsson *et al*. used a FFQ specifically designed to capture Cd exposure from both food and beverage items (including water)[20]. The FFQ used by Adams *et al*. from the Women’s Health Initiative included 122 individual foods or food groups, frequency and portion information [15, 21] referring to the previous 3 months. Julin *et al*. assessed D-Cd using a 67-item FFQ which was validated against a 1-week weighted diet record for intake of cadmium rich foods (whole grain bread, breakfast cereals, potatoes, root crops, cabbage, and spinach) [22]. Quraishi *et al*. obtained U-Cd concentration and D-Cd estimates from 1,050 women from the Women Health Initiative (WHI), with an average age of 63.4 years [18]. All of these studies reported a weak correlation between FFQ-estimated D-Cd and U-Cd, but only the Quraishi study provided stratified analysis by smoking status, highlighting the need to further investigate the concordance between estimated and measured exposure in different study populations. This is of particular importance since FFQ-based Cd intake estimates have been used to assess hazard risk ratios of health outcomes in the general population [17, 23–27].

In this study we test the comparability of D-Cd, as estimated via the FFQ used in the Danish Diet Cancer and Health Cohort [28], and measured U-Cd levels, normalized by creatinine, in a cohort of 1,764 Danish post-menopausal women that included never, former and current smokers.

## Materials and Methods

### Ethics Statement

The present study was approved by the regional research ethic committee for Copenhagen and Frederiksberg. Written informed consent was obtained from all study participants upon enrolment into the cohort. The present analysis was carried out without contact to the cohort members or their families. Anonymity of participants was retained by strict data management.

### Study Population

From December 1, 1993, through May 31, 1997, a total of 57,053 individuals (29,875 women and 27,178 men), who were aged 50–65 years, born in Denmark, and had no previous cancer diagnosis, were enrolled in the prospective Diet, Cancer and Health cohort [29]. At enrolment, each participant gave a sample of urine and completed a self-administered, interviewer-checked 192 item semi-quantitative FFQ and a questionnaire covering lifestyle habits, including information on smoking history, reproductive history, health status, and social factors. Smoking intensity corresponded to the reported numbers of cigarettes smoked daily, while pack-years was calculated as the number of 20-cigarette packs smoked daily, multiplied by the years of smoking. Exposure to second-hand smoke was reported as a categorical variable to reflect exposure to cigarette smoke either in the household or at work, starting from age 30 and above. In total 56,999 persons filled in the detailed dietary questionnaires. Of these, we obtained U-Cd levels from 1,764 postmenopausal women who were selected to be part of a case-cohort study on Cd and breast cancer [24]; 896 women developed breast cancer from 4 years after baseline visit and through 2012, and 868 women did not develop breast cancer between baseline and 2012. Case status will be considered in the analyses presented.

### Dietary Cadmium and Nutrient Intake Estimates

Daily dietary intake of calories (kcal day^-1^), cadmium (D-Cd, ngn day^-1^), zinc (Zn) and iron (Fe) (mg day^-1^) were obtained for each participant based on the 192 item semi-quantitative FFQ filled out at enrolment, as previously described [24, 28, 30–33]. Dietary intake estimates of Zn and Fe were obtained by summing estimates from food and supplement consumption. Since D-Cd intake estimates and the daily caloric intake were highly correlated, D-Cd estimates were normalized bycaloric intake (ng kcal^-1^), according to the nutrient density approach described by Willet *et al*. [34].n, according to the nutrient density approach described by Willet *et al*. The Danish Food Monitoring Programme for Nutrients and Contaminants, 1993–97, along with the FFQ, were used to estimate individual daily cadmium intake [35]. The 5-year monitoring period 1993–97 was used, since it matches with the enrolment period of the DCH cohort. Estimates of Cd, Fe and Zn content were obtained for over 80 food items commonly available on the Danish market.

### Urinary Cadmium Levels

Cd was measured inurine samples, which wereere collected in transparent polypropylene cups (USON Plast, Denmark) and stored in transparent 1 ml polypropylene cryotubes (NUNC, Denmark). The urine was never in contact with any metallic equipment, and the suppliers guaranteed that Cd was not used in the manufacturing of collection and storage materials. Urinary cadmium concentrations were determined using a Thermo X-Series 2 (Bremen, Germany) inductively coupled plasma mass spectrometer (ICP-MS) following digestion in the presence of high-purity acids and oxidants in a Class 100 clean hood to prevent contamination by atmospheric particulates. Urinary creatinine concentrations were quantified using a Cayman Chemicals Creatinine Assay Kit No. 500701 (Cayman Chemicals, Ann Arbor, MI, USA) with UV-VIS measurement at 500 nm employing a Beckman Coulter DU800 UV/VIS Spectrometer (Beckman Instruments Inc., Brea, CA, USA). Samples below the limit of detection (LOD) (N = 23) were assigned to the batch Cd LOD √2^−1^[36, 37]. For each urine sample, Cd concentration (μg L^-1^) was divided by the determined creatinine amount (g L^-1^) to obtain creatinine-adjusted urinary cadmium levels (U-Cd) in μg Cd g creatinine^-1^. LOD varied between <0.0011 and 0.047 μg/L across 30 discrete analytical batches. Average recovery of NIST standard reference material (SRM) 2668was 90.2%, across all analysis batches. The lower and higher levels of Cd in the NIST-SRM 2668 were 1.06 ± 0.05 and 16.40 ± 0.25 μg/L. Random incurred sample re-testing of 5% of the samples showed Pearson correlation *r* = 0.90 with initial measurements.

### Statistical Analyses

Spearman’s r coefficient and p value were calculated for creatinine-adjusted U-Cd and D-Cd. The ANOVA test wasas used to identify differences in the mean value of Cd measures (either D-Cd estimates or log-transformed creatinine-adjusted U-Cd) across categories of 10 covariates (creatinine-adjusted U-Cd, D-Cd, smoking status, pack-years, caseca status, energy intake, BMI, age, iron intake and parity).

Linear regression models were used to investigate the association between D-Cd, as the independent variable, and creatinine-adjusted U-Cd, as the dependent variable. Models were adjusted for *a priori* defined potential covariates: age, smoking status (never, former, current), pack-years (packs-per-year and years smoking), and Fe intake (mg Fe day^-1^). Regression analyses were run for all participants and stratified by smoking status. We used an interaction term to test if the association between D-Cd and U-Cd differed by age, sex, pack-years, daily energy intake, BMI, daily Zn and Fe intake.

An unsupervised forward selection procedure was used to explore the relevance of specific dietary and demographic items as predictors of urinary Cd levels. Briefly, we constructed general linear models with continuous log-transformed creatinine-adjusted U-Cd as a dependent variable for never, former and current smokers and for all participants. A model optimization procedure was used that added variables at each step that provide the greatest value of the adjusted R^2^ statistic, stopping at the step where the significance level corresponding to the addition of a predictor was greater than 0.2. The predictor variables available to the model selection procedure were: red meat (g day^-1^), soy (g day^-1^), all fish and seafood (g day^-1^), fruit (g day^-1^), all meat (g day^-1^), potatoes (g day^-1^), vegetables and fruit (g day^-1^), fruits and grains (g day^-1^), all grains (g day^-1^), leafy vegetables (g day^-1^), mushrooms (g day^-1^), whole grains (g day^-1^), white grains (g day^-1^), tea (g day^-1^), wine (g day^-1^), beer (g day^-1^), spirits (g day^-1^), Fe (mg day^-1^) and Zn (mg day^-1^), age (years), BMI (kg m^-2^), smoking duration (years, for former smokers only), number of children (0 (reference), 1–2, ≥3), D-Cd (μg day^-1^), D-Cd normalized by body weight (μg day^-1^ kg^-1^), D-Cd normalized by daily caloric intake (ng kcal^-1^), smoking status (never, former, current), later development of breast cancer (no, yes), pack-years (n. of packs day^-1^ years of smoking, for current smokers model only), exposure to second-hand cigarette smoke at work or home since 50 years of age (yes or no, for never smokers only), number of decades of exposure to second-hand cigarette smoke at work or home since 40 years of age (0 to 4, for never smokers only) and average daily caloric intake (kcal day^-1^). The food items included in the selection procedure were chosen based on known literature [24, 38, 39] and information contained in the ATSDR Cadmium Toxic Substance Portal [40]. We were not able to consider second-hand smoke among smokers because of collinearity with smoking status. In order to exclude the possibility that the selected variables would be selected because of association with creatinine, rather than with U-Cd [41], we also used the unsupervised forward selection procedure to construct models using ln(U-Cd) as the dependent variable, and added urinary creatinine (g L^-1^) as a predictor instead.

Creatinine-adjusted U-Cd was transformed by the natural logarithm to account for non-normal distribution. The *β* estimates were natural-logarithm back transformed, followed by subtractingsubtracting 1 and multiplyingying by 100 [(e^β^-1)*100], obtaining the % change in the dependent variable U-Cd per a 1-unit change in the independent variables listed. The procedure PROC GLMSELECT was used for the model optimization procedure, specifying the option “selection = forward (select = ADJRSQ stop = SL SLE = 0.2)”. All analyses were performed in SAS version 9.3 (SAS Institute, Cary, North Carolina, USA).

For each regression model, we have reported R^2^ adjusted by degrees of freedom [42], *β* values, and p values.

## Results

Demographic characteristics and descriptive statistics of our study population are summarized in Table 1. U-Cd mean ± SD was found to be 0.46 ± 0.60 μg Cd L^-1^ without creatinine adjustment and 0.70 ± 0.62 μg Cd g creatinine^-1^, while D-Cd mean ± SD was 14.00 ± 4.35 (ngn Cd day^-1^).

**Table 1:** Demographic characteristics of study population (*n* = 1764).

Variable (units)	Unit	Mean ± SD	Median (5%, 95%)
U-Cd	(μg L^-1^)	0.46 ± 0.60	0.27 (0.03, 1.48)
Urinary creatinine	(mg L^-1^)	691.83 ± 553.42	536.38 (121.37, 1750.00)
Creatinine-adj. U-Cd	(μg Cd g creatinine^-1^)	0.70 ± 0.62	0.52 (0.14, 1.85)
D-Cd	(μg Cd day^-1^)	14. ± 4.44	13.5 (7.88, 21.88)
Calories-adj. D-Cd	(ngn Cd kcal^-1^)	6.8 ± 1.44	6.7 (4.77, 9.22)
Age	(years)	57.02 ± 4.26	57.00 (51.00, 64.00)
BMI	(kg m^-2^)	25.71 ± 4.32	24.98 (20.22, 33.64)
Energy intake	(kcal day^-1^)	2080.62 ± 549.78	2022.63 (1317.52, 3039.62)
Pack-years^a^	(packs day^-1^ years-smoking)	26.72 ± 15.73	25.85 (4.40, 51.00)
Smoking intensity^a^	(n. cigarettes day^-1^)	14.91 ± 7.47	15.00 (4.00, 25.00)
Dietary Iron	(μg Fe day^-1^)	16.43 ± 11.60	13.57 (7.46, 29.12)
Dietary Zinc	(μg Zn day^-1^)	17.33 ± 8.19	14.72 (8.20, 30.96)

^a^ For current smokers only, *n* = 584.

As can be seen in Table 2, as U-Cd increased, D-Cd decreased. This inverse relation was confirmed by a negative rank correlation coefficient (Spearman *r* = -0.14, *p* = <0.0001) for the entire population. The correlation was positive, but weak and borderline significant, in never smokers (Spearman *r* = 0.07, *p* = 0.06). As expected, never smokers exhibited lower levels of creatinine-adjusted U-Cd than both former and current smokers (ANOVA *p* <0.01, Table 2), and U-Cd levels were higher in current smokers than former smokers and correlated toto increasing pack-years (ANOVA *p* <0.01). Conversely, D-Cd was lower in current smokers than in never smokers. Higher levels of U-Cd were found in older women and also increased with children parity (ANOVA *p* <0.01, Table 2). U-Cd was marginally lower in individuals with higher Fe intake while D-Cd was higher in those with increased Fe intake and lower in those with greater energy intake. Participants that reported exposure to second-hand smoke had lower U-Cd and D-Cd values. There was no difference in U-Cd or D-Cd levels observed between cancer cases and controls, or BMI ranges.

**Table 2:** Mean ± Standard Deviation (SD) of U-Cd (μg Cd g creatinine^-1^) and D-Cd (ng Cd kcal^-1^) across cohort characteristics.

Group	Range	*n* (%)	U-Cd		D-Cd	

			Mean ± SD	ANOVA^b^	Mean ± SD	ANOVA^b^
Creatinine-adj. U-Cd	<0.31	449 (25%)	-	-	6.89 ± 1.30	**<0.01**
(μg g creatinine^-1^)	0.31–0.52	424 (24%)	-	-	6.87 ± 1.40	
	0.52–0.89	451 (26%)	-	-	6.77 ± 1.33	
	>0.89	440 (25%)	-	-	6.58 ± 1.47	
D-Cd	<5.82	442 (25%)	0.83 ± 0.73	**0.04**	-	-
(ng kcal^-1^)	5.82–6.78	487 (28%)	0.67 ± 0.58	** **	-	-
	6.78–7.62	393 (22%)	0.63 ± 0.54	** **	-	-
	>7.62	442 (25%)	0.65 ± 0.59	** **	-	-
Smoking status	Never	782 (44%)	0.47 ± 0.41	**<0.01**	6.93 ± 1.31	**<0.01**
	Former	398 (22%)	0.67 ± 0.57	** **	7.05 ± 1.38	
	Current	584 (33%)	1.02 ± 0.73	** **	6.39 ± 1.40	
Second-hand smoke (> 50 years old)^d^	No	241 (31%)	0.50 ± 0.53	0.12	7.19 ± 1.27	**<0.01**
	Yes	541 (69%)	0.45 ± 0.34		6.82 ± 1.31	
Pack-years^a^	<19	192 (33%)	0.74 ± 0.47	** **	6.70 ± 1.50	**<0.01**
(packs day^-1^ years-smoking)	19–32	192 (33%)	1.09 ± 0.76	** **	6.37 ± 1.17	
	>32	200 (34%)	1.23 ± 0.82	**<0.01**	6.09 ± 1.43	
Breast cancer status	Non-case	868 (49%)	0.72 ± 0.67	** **	6.82 ± 1.45	0.13
	Case	896 (51%)	0.67 ± 0.55	** **	6.75 ± 1.30	
Energy intake	< 1805	577 (33%)	0.68 ± 0.58	0.73	6.97 ± 1.50	**<0.01**
(kcal day^-1^)	1805–2240	577 (33%)	0.73 ± 0.65		6.77 ± 1.31	
	> 2240	610 (34%)	0.68 ± 0.62	0.36	6.60 ±1.30	
BMI	<25^c^	887 (50%)	0.73 ± 0.69		6.81 ± 1.43	0.53
(kg m^-2^)	25–30	625 (35%)	0.67 ± 0.54		6.75 ± 1.32	
	>30	249 (14%)	0.68 ± 0.53	0.85	6.71 ± 1.37	
Age	<54	580 (33%)	0.65 ± 0.63		6.82 ± 1.44	0.71
(years)	54–59	601 (34%)	0.71 ± 0.63		6.76 ± 1.41	
	>59	583 (33%)	0.73 ± 0.58	**<0.01**	6.76 ± 1.30	
Fe intake	<11.28	581 (33%)	0.73 ± 0.67		6.60 ± 1.38	**<0.01**
(μg day^-1^)	11.28–16.90	585 (33%)	0.70 ± 0.61	** **	6.85 ± 1.35	
	>16.90	598 (34%)	0.66 ± 0.57	0.19	6.88 ± 1.40	
Parity	Null	246(14%)	0.67 ± 0.62		6.93 ± 1.43	0.16
(n. of children)	1–2	1063(59%)	0.67 ± 0.58	** **	6.76 ± 1.36	
	3 or more	455(26%)	0.76 ± 0.69	**<0.01**	6.73 ± 1.40	

p<0.05 were highlighted in bold

^a^Current smokers only (*n* = 584)

^b^ANOVA p value for the normal D-Cd and log transformed U-Cd

^c^Includes normal and underweight BMI

^d^Never smokers only (*n* = 782).

The non-adjusted association between creatinine-adjusted U-Cd and D-Cd in a regression analysis was small (R^2^ = 0.007), negative (*β* = -4.79% change in U-Cd per unit increase of D-Cd), and significant (*p* < 0.01) in the whole study population (Table 3). However the association disappeared (*p* = 0.67) when the model was controlled for age, smoking status, and Fe intake, with smoking status explaining the largest proportion of the variance. In adjusted models, the association between creatinine-adjusted U-Cd and D-Cd was positive in never smokers (*β* = 5.13% change in U-Cd per unitunit increase of D-Cd, *p* < 0.01) and negative in current smokers (*β* = -5.08% change in U-Cd per unit increase of D-Cd, *p* = 0.01) (Table 3). However, the overall R^2^ values in these cases remained small. D-Cd was not associated with U-Cd in former smokers. D-Cd was not associated with U-Cd in adjusted models when the study population was stratified into tertiles of age or Fe intake, or by disease status, and there was no evidence of interaction between D-Cd and age, Fe, or disease (data not shown).

**Table 3:** Associations between D-Cd (ng kcal^-1^) and creatinine-adj. U-Cd^d^ (μg Cd g creatinine^-1^).

Smoking	Model	R^2^ ^a^	% change^b^	p value
All	Unadj.	0.0068	-4.79	**<0.01**
(*n* = 1764)	Adj.^c^	0.2372	0.53	0.67
Never smokers	Unadj.	0.0055	4.49	**0.02**
(*n* = 782)	Adj.^c^	0.0565	5.13	**<0.01**
Former smokers	Unadj.	-0.0024	0.49	0.86
(*n* = 398)	Adj.^c^	0.0576	2.12	0.43
Current smokers	Unadj.	0.0184	-6.85	**<0.01**
(*n* = 584)	Adj.^c^	0.0945	-5.08	**<0.01**

p <0.05 were highlighted in bold

^a^R^2^ adjusted by degrees of freedom

^b^Percent change for creatinine-adj. U-Cd for a one ng kcal^-1^ increase of D-Cd was calculated as (e^β^-1)*100 n100

^c^Each model was adjusted for age, smoking status (in the all population only), BMI, pack-years (in current smokers only) and iron intake

^d^U-Cd is natural-log transformed.

A forward model selection procedure, aimed to identify factors predictive of U-Cd, selected different combinations of 17 out of 36 possible predictor variables across 4 different models (Table 4). The maximum R^2^ obtained in the entire population was 0.22, and was achieved with a combination of 9 variables that included (in order of decreasing R^2^ magnitude) being current or former smoker, age, consuming tea, soy, or wine, having 3 or more children, consuming red meat or vegetables/fruit products. In never smokers, the optimal fit included 10 predictor variables (age, vegetables/fruit, red meat, soy, wine, fruit, fish, whole grains, having 3 or more children and spirits), resulting in an R^2^ of 0.09. In former smokers, the model selection procedure produced an optimal R^2^ of 0.09 using a combination of smoking duration, wine, having 3 or more children, soy, age and Zn. In current smokers, a maximum R^2^ of 0.10 was obtained from a model including a combination of 7 predictor variables, including pack-years, tea, soy, BMI, age, D-Cd and energy intake. The variables that generally contributed the most to the R^2^ in the different models were smoking status (for the general population), age (for never smokers), smoking duration (for former smokers), and pack-years (for current smokers). A higher number of childbirths was consistently associated with increasing levels of U-Cd. In terms of dietary contributors, soy product consumption was consistently associated with higher U-Cd with the largest estimates across all models (β ≥0.20% change in U-Cd per each g day^-1^ increase of soy consumption). Wine was negatively associated with U-Cd (*β* max. -0.06% decrease in U-Cd per each g day^-1^ increase of wine consumption). No other food variable was consistently associated with U-Cd in all models. Consumption of red meat, wine and tea appeared consistently inversely associated with U-Cd, although their contributions were generally small and not always significant (*β* < -0.3% change in U-Cd per unit increase of either item consumption). Dietary Zn was marginally inversely correlated with U-Cd in former smokers (*β* = -0.76% change in U-Cd per mg Zn day^-1^); dietary Fe was not selected in any models as a significant contributor to U-Cd levels. When we used non-normalized ln(U-Cd) as dependent variable, and added urinary creatinine to the predictors, we obtained very similar results in all selection models, with the main difference that the best predictor of U-Cd levels was creatinine itself.

**Table 4:** Forward model selection to predict creatinine-adj. U-Cd^a^ (μg Cd g creatinine^-1^). Parameter estimates are listed for the factors selected by the procedure for inclusion in each model (p value cut-off = 0.2).

Model effects	All			Never smokers			Former smokers			Current smokers		
	(*n* = 1764)			(*n* = 782)			(*n* = 398)			(*n* = 584)		
	ΔR^2^ ^b^	% change^c^change	p value	ΔR^2^ ^b^	% change^c^change^c^	p value	ΔR^2^ ^b^	% change^c^change^c^	p value	ΔR^2^ ^b^	% change^c^change^c^	p value
Dietary Cd (ng kcal^-1^)										<0.01	-3.93%	0.10
Age (years)	0.02	2.02%	<0.01	**0.05**	4.11%	<0.01	<0.01	1.45%	0.08	<0.01	1.15%	0.09
BMI (kg m^-2^)										<0.01	-1.39%	0.06
Current smoker	**0.17**	125%	<0.01									
Former smoker	0.03	35%	<0.01									
Smoking duration (years)		** **					**0.05**	1.55%	<0.01			
Pack-years (packs day^-1^ years-smoking)										**0.07**	1.08%	<0.01
Childbirth >3 (categorical)	<0.01	11.16%	0.04	<0.01	14.86%	<0.01	0.01	19.22%	<0.01			
Vegetables/fruit (g day^-1^)	<0.01	0.01%	0.14	0.01	0.06%	<0.01						
Red meat (g day^-1^)	<0.01	-0.11%	0.13	0.01	-0.28%	0.01						
Fish (g day^-1^)				<0.01	-0.23%	0.08						
Soy (g day^-1^)	<0.01	0.36%	<0.01	0.01	0.44%	0.01	0.01	0.22%	0.03	<0.01	0.31%	0.06
Wine (g day^-1^)	<0.01	-0.01%	0.02	0.01	-0.04%	0.05	0.01	-0.06%	0.01	<0.01	-0.03%	0.15
Whole grain (g day^-1^)				<0.01	0.07%	0.09						
Fruit (g day^-1^)				<0.01	-0.05%	0.07						
Spirits (g day-1)				<0.01	-0.49%	0.19						
Tea (g day^-1^)	<0.01	-0.01%	<0.01							0.01	-0.02%	<0.01
Zinc (mg day^-1^)							<0.01	-0.76%	0.08			
Total model Adj. R^2^	0.22			0.09			0.09			0.10		

^a^U-Cd is natural-log transformed

^b^ Percent change for creatinine-adj. U-Cd for a one unit increase of each independent variable was calculated as (e^β^-1)*100

^c^R^2^ adjusted by degrees of freedom.

The overall concordance across quartiles was 26%, not dissimilar from what would be obtained by chance (S1 Table).

## Discussion

We report a weak association between measured U-Cd and dietary estimates of Cd in this cohort of 1,764 post-menopausal Danish women. This association was found to be positive among never smokers and negative among current smokers in adjusted regression models. Our U-Cd and D-Cd results were in line with levels previously measured in non-occupationally exposed populations and were below the internationally established safety thresholds [11, 13, 40].

Since smoking is a major source of cadmium exposure, we hypothesized that never smokers are most likely to show the strongest association between D-Cd and U-Cd. Our results confirmed our hypothesis and showed a significant positive association between D-Cd and U-Cd in never smokers. The adjusted R^2^, however, was small, suggesting that overall the intake estimates are of limited use to approximate cumulated Cd uptake, even in never smokers. In contrast, we found a small, significant, and negative, association between D-Cd and U-Cd in current smokers. This could be explained by lower consumption of foods rich in cadmium, such as grains and vegetables, among smokers [43, 44]. Consistent with the current understanding that U-Cd reflects long-term Cd storage in the kidneys [8, 15, 19], yet in contrast to reports by Chaumont *et al*., 2013 and Paschal *et al*., 2000 [45, 46], we found significantly higher levels of U-Cd in former smokers than in never smokers. We also found that age is a strong predictor of U-Cd in never smokers, consistent with previous reports [9, 16, 45], which is especially striking since our cohort included only postmenopausal women within a limited age range. Second-hand smoke was not associated with U-Cd among never smokers, suggesting that passive smoke was not a sizable contributor to U-Cd levels in our population.

Childbirth was positively associated with U-Cd levels, which can be explained by depleted Fe stores during pregnancy [47]. Lower body stores of divalent cations (specifically Fe and Zn) have been implicated in the absorption and toxicity of Cd [48]. However our variables for Fe or Zn intake were not consistently selected as U-Cd determinants during the selection procedure across models (Table 4). This observation may be explained by the fact that after menopause, Fe body stores increase [49] or simply by the higher uncertainty associated with estimating Fe and Zn levels from dietary sources, rather than measuring blood Zn and Fe content [22].

We found that soy was the most significant dietary predictor of U-Cd, consistent with a previous finding [15], even though the consumption of soy in our cohort was quite low (on average less than 1g day^-1^). Consumption of tea and red meat were generally associated with lower levels of U-Cd, although only marginally. An inverse relation between Cd in blood and total meat consumption was also reported by Bjermo *et al*. [48]. An inverse correlation was found with wine as well, andand while the percent change per gram appears modest, each glass of wine contains approximately 148 g resulting in -8.9% U-Cd per daily glass among former smokers.

The correlation between estimated D-Cd and measured U-Cd in never smokers (r = 0.07) was much smaller than what was reported by measuring Cd directly in four days of food duplicates of never smokers (r = 0.38–0.43) and correlating it with U-Cd in spot urine samples [22, 50]. That report is somewhat surprising since U-Cd is thought to be a marker of long-term cumulative exposure, and therefore one might think that it should not correlate as well with measured Cd from recently collected duplicate food samples. The authors, Julin *et al*. (2011), argue that the dietary samples collected reflect long-term food preferences [22], which is a possible explanation for the substantial correlation between Cd in the food duplicates and measured U-Cd. Another explanation might be that the influence of acute exposure on U-Cd may not be tiny. While some studies have reported low intra-individual temporal variability in U-Cd (ICC = 0.7–0.9) [51, 52], others suggest it may be larger (ICC~0.5) [9, 53, 54]., others suggest it may be larger (ICC~0.5) The smaller correlation between FFQ-derived D-Cd and measured U-Cd in never smokers (r = 0.07) in this study, therefore, mayay reflect uncertainties in the FFQ-derived Cd intake estimate or intra-individual variability in the U-Cd measure.

While not perfect, FFQs have been validated for estimating energy, macronutrients (protein, fat, sodium and potassium), and some micronutrients [21, 28, 55, 56]. Energy adjustment is essential in FFQ-derived estimates because estimates of nutrients and contaminants are often highly correlated with energy intake [34], as they were here., as they were here. In this study we used the nutrient density approach dividing D-Cd by total calorie intake; a sensitivity analysis using the residual approach produced highly similar results. FFQs are seldom validated against other dietary assessment methods for dietary contaminants, and to our knowledge have not been validated for D-Cd. Regional variability in cadmium levels in foodstuffs also likely contributes to errors in the FFQ-derived D-Cd estimates [57]. In addition, exposure estimates from FFQs are known to be influenced by recall bias and inconsistencies in portion size reporting, especially if it is administered only one time [58, 59]. The results from our current study and previously published studies [15–18, 22] suggest that D-Cd from an FFQ has limited value as a predictor of U-Cd.

Recent epidemiologic studies have reported on the association between D-Cd from FFQs and several health outcomes [17, 24, 25, 27, 60–62]. The majority of the studies have reported null findings, and have suggested that one of the reasons may be non-differential exposure misclassification which tends to attenuate results toward the null [59], resulting from the FFQ-derived D-Cd estimate. While our results cannot confirm this error in the FFQ-derived D-Cd estimate, we clearly show a lack of association with U-Cd, a good biomarker of long-term exposure [11, 12], suggesting that non-differential exposure misclassification may be contributing to the epidemiologic findings. On the other hand, it is possible that while U-Cd is strongly correlated with Cd stored in the kidneys [63], in whichCd has an estimated half-life of ~45 years [64], it, it may not reflect doses of relevance in other organs.

The design and performance of this study has multiple strengths. First, it is the largest study investigating concordance between U-Cd and FFQ-estimated dietary Cd exposure. It relies on a validated FFQ of over 192 food items and recipes [28]; this thorough study design allowed us to investigate the association of Fe and Zn intake with U-Cd. There are large numbers of women among different categories of smoking status, to allow us to account for the role of smoking on the association between D-Cd and U-Cd. The trace inorganics laboratory used a robust analytical method with good standard reference material recoveries, a very low detection limit for U-Cd, and displayedgood reproducibility in the U-Cd measure. This study which enrolled a large sample of Danish citizens allowed for applying dietary Cd estimates from food basket survey and typical recipes to a relatively homogeneous population.

The limitations of this study must be carefully considered when interpreting the results. The Danish Diet Cancer and Health subcohort utilized in this study was not a fully representative sample of Denmark women, given the limited age range, and selection of only post-menopausal women [24]. Additionally, approximately 50% of this study population developed breast cancer 4+ years after urine collection and FFQ completion; although results did not differ when cancer cases were excluded from the analysis. Another limitation is the lack of a standardized collection time for the spot urine collection. First-morning urine and 24-h collection are generally considered better samples to estimate U-Cd [52]. Another limitation is the lack of a standardized collection time for the spot urine collection. First-morning urine and 24-h collection are generally considered better samples to estimate U-Cd.

We also did not include information about toxicokinetics in our study. Toxicokinetic models have been developed to approximate Cd body burden based on dietary intake [11, 12], however when duplicate diet D-Cd was entered in such a toxicokinetic model, derived from the Nordberg-Kjellström equation [22, 65], the D-Cd–U-Cd correlation increased only modestly (from r = 0.43 to r = 0.54) despite adding the calculated intestinal absorption rate and mobilization to the model [11]. These results suggest that the duplicate diet component is more important than the toxicokinetic component in correlating D-Cd with U-Cd.

## Conclusions

We found only weak association between FFQ-derived dietary intake estimates and urinary levels of cadmium in this large older female population. Estimated dietary cadmium intake from FFQ likely has limited use in epidemiological studies.

## Supporting Information

S1 Table Cross classification of D-Cd and creatinine-adjusted U-Cd in never smokers (*n* = 782). D-Cd/U-Cd column sum, (column %). (DOCX)

## Abbreviations

U-Cd: urinary cadmium
D-Cd: dietary cadmium
Fe: iron
Zn: zinc
BMI: body mass index
