# Supplementary material for: Dietary Intake Estimates and Urinary Cadmium Levels in Danish Postmenopausal Women
Source: PLoS One. 2015 Sep 21;10(9):e0138784. doi: 10.1371/journal.pone.0138784 (PMC4577120; doi:10.1371/journal.pone.0138784)
Supplement: S1 Table — (DOCX) [file pone.0138784.s001.docx]

**Dietary Intake Estimates and Urinary Cadmium Levels in Danish Postmenopausal Women**

Caterina Vacchi-Suzzi^1^, Kirsten T. Eriksen^2^, Keith Levine^3^, Jane McElroy^4^, Anne Tjønneland^2^, Ole Raaschou-Nielsen^2, 5^, James M. Harrington^3^ and Jaymie R. Meliker^1^

^1^ Department of Preventive Medicine and Program in Public Health, Stony Brook University, Stony Brook, NY, USA

^2^ Danish Cancer Society Research Center, Copenhagen, Denmark

^3^ RTI International Trace Inorganics Department, Research Triangle Park, NC, USA

^4^ Family and Community Medicine, University of Missouri, Columbia, MO, USA

^5^ Department of Environmental Science, Aarhus University, Aarhus, Denmark

**Supplementary Results**

Supplementary Table 1. Cross classification of D-Cd and creatinine-adjusted U-Cd in never smokers (*n* = 782). D-Cd/U-Cd column sum, (column %).

| Cross-classification | First U-Cd quartile | Second U-Cd quartile | Third U-Cd quartile | Fourth U-Cd quartile |
| --- | --- | --- | --- | --- |
| n (%) in first D-Cd quartile | **53/200 (27%)** | 56/194 (29%) | 46/191 (24%) | 39/197 (20%) |
| n (%) in fourth D-Cd quartile | 42/200 (21%) | 50/194 (26%) | 51/191 (27%) | **53/197 (27%)** |
